# DDT and Breast Cancer: Cohn et al. Respond

**DOI:** 10.1289/ehp.11025R

**Published:** 2008-04

**Authors:** Barbara A. Cohn, Piera M. Cirillo, Robert I. Sholtz, Mary S. Wolff

**Affiliations:** Center for Research on Women’s and Children’s Health, Public Health Institute, Berkeley, California, E-mail: bcohn@chdstudies.org; Mount Sinai School of Medicine, New York, New York

We thank Tarone for his letter, as it provides an opportunity to elaborate on analytic strategies for the study of DDT associations with breast cancer.

One feature of our study (Cohn et al. 2007)—assessment of exposure in blood samples collected during active DDT use in the 1960s—provided a unique opportunity to examine three DDT-related compounds singly and in combination. The three DDT-related compounds studied represent distinct aspects of exposure. *p,p*′-DDT [1,1,1-trichloro-2,2-bis(*p*-chlorophenyl)ethane] is the primary ingredient of commercial grade DDT. *p,p*′-DDE [1,1-dichloro-2,2-bis(*p*-chlorophenyl)ethylene], the most persistent DDT-related compound, is a metabolite of *p,p*′-DDT that is both made by humans during active exposure, and also ingested directly from food sources where it can be stored for long periods in fat (Morgan and Roan 1975). *o,p*′-DDT [[1,1,1-trichloro-2(*p*-chlorophenyl)-2-(*o*-chlorophenyl)ethane] is a low-concentration contaminant of commercial DDT that is eliminated by humans most quickly, making it a marker of recent exposure (Morgan and Roan 1975). Therefore, absolute and relative DDT/DDE isomer levels may represent different windows of exposure (Wolff et al. 2007).

Unlike our investigation, most other breast cancer studies were conducted long after active use of DDT ceased. Thus the preponderance (> 95%) of their exposure was only *p,p*′-DDE [see our Figure 1 and Table 1 (Cohn et al. 2007)]. Hence, our study provides new information. An additional dimension is that these compounds have been shown to have distinctly different endocrine activity (Kelce et al. 1995), suggesting potential for differential effects on human outcomes. Therefore, we disagree with Tarone’s assertion that the lack of an association between *p,p*′-DDE and breast cancer risk in young women refutes a role for *p,p*′-DDT exposure. The timing, origin, and functional activity may differ for each compound.

Concurrent measurements of *p,p*′-DDT, *p,p*′-DDE, and *o,p*′-DDT allow evaluation of potential differences in the effects of these compounds. Other studies have also observed differing associations with cancer risk for *p,p*′-DDT and its metabolite, *p,p*′-DDE. McGlynn et al. (2006) reported that the *p,p*′-DDT association with risk of liver cancer was enhanced when *p,p*′-DDE was low. We also reported that a higher proportion of *p,p*′-DDT to *p,p*′-DDE in maternal serum samples was associated with longer time to pregnancy in their daughters 30 years after exposure *in utero* (Cohn et al. 2003). In another other breast cancer study, Romieu et al. (2000) showed a significant effect for *p,p*′-DDE—after adjustment for *p,p*′-DDT—for predicting breast cancer, particularly in postmenopausal women. We believe that simultaneous adjustment for DDT-related compounds is a strength of our study.

Tarone suggests that subgroup analyses weaken the results of our article (Cohn et al. 2007). However, we pointed out in our article that subgroup analyses, by birth cohort, were planned *a priori* and were a primary objective of our study. In this setting, subgroup analyses are a strength that enabled us to examine whether age at DDT exposure may be of importance in human breast cancer.

The trends in breast cancer incidence in young women previously presented by Tarone in Table 1 of his article (Tarone 2006) do not refute a possible effect of DDT exposure in childhood. Successive birth cohorts of women diagnosed at 20–39 years of age between 1975 and 2002 (Table 1; Tarone 2006) experienced decreasing DDT exposure in childhood (birth years 1941–1982) because DDT use began in 1945, peaked in 1959, and was banned in 1972 in the United States (U.S. Environmental Protection Agency 1975). Successive birth cohorts of women diagnosed at 40–49 years of age between 1990 and 2002 (Table 1 in Tarone 2006) were all exposed to DDT in childhood (birth years 1941–1962); therefore, breast cancer trends for these birth cohorts are not informative for investigating effects of DDT exposure in childhood. Further, we agree with Weiss (2007) that trends in invasive disease and mortality cannot be interpreted without consideration of the rising incidence of *in situ* disease and its successful treatment, which would reduce incidence of invasive disease and mortality.
